# A Hiatus of the Greenhouse Effect

**DOI:** 10.1038/srep33315

**Published:** 2016-09-12

**Authors:** Jinjie Song, Yuan Wang, Jianping Tang

**Affiliations:** 1 School of Atmospheric Sciences, Nanjing University, China

## Abstract

The rate at which the global average surface temperature is increasing has slowed down since the end of the last century. This study investigates whether this warming hiatus results from a change in the well-known greenhouse effect. Using long-term, reliable, and consistent observational data from the Earth’s surface and the top of the atmosphere (TOA), two monthly gridded atmospheric and surface greenhouse effect parameters (*G*_*a*_ and *G*_s_) are estimated to represent the radiative warming effects of the atmosphere and the surface in the infrared range from 1979 to 2014. The atmospheric and surface greenhouse effect over the tropical monsoon-prone regions is found to contribute substantially to the global total. Furthermore, the downward tendency of cloud activity leads to a greenhouse effect hiatus after the early 1990 s, prior to the warming pause. Additionally, this pause in the greenhouse effect is mostly caused by the high number of La Niña events between 1991 and 2014. A strong La Niña indicates suppressed convection in the tropical central Pacific that reduces atmospheric water vapor content and cloud volume. This significantly weakened regional greenhouse effect offsets the enhanced warming influence in other places and decelerates the rising global greenhouse effect. This work suggests that the greenhouse effect hiatus can be served as an additional factor to cause the recent global warming slowdown.

The rate at which the global average surface air temperature (*T*_*s*_) increases has slowed down during the past few decades^1^. This so-called hiatus, pause, or slowdown of global warming has inspired investigations into its potential causes worldwide^1^,^2^. Although some researchers doubted the existence of a global warming hiatus because of coverage bias^3^,^4^, artificial inconsistency^5^, and a change point analysis of instrumental *T*_s_ records^6^, it is now accepted that a recent warming deceleration can be clearly observed^1^. There are two primary hypotheses to explain the recent slowdown of the upward trend in *T*_*s*_^7^. Both hypotheses attempt to explain the contradiction between the trendless *T*_*s*_ variation and the intensifying anthropogenic greenhouse effect resulting from the steadily increasing emission of greenhouse gases (GHGs). The first attributes the warming hiatus to external radiative forcings, such as decreasing solar irradiance^8^, increasing tropospheric and stratospheric aerosols^9^, reduced stratospheric water vapor^10^, and several small volcanic eruptions^11^. The warming effect of increasing GHGs is largely cancelled out by the decreasing solar shortwave radiation received by the Earth’s surface. The second considers the warming pause to be a result of internal oceanic and/or atmospheric decadal variabilities against the centennial warming trend^12^, in which two leading theories are proposed. One asserts that the recent warming hiatus likely results from a La Niña-like state or a negative phase of Interdecadal Pacific Oscillation (IPO) associated with the cooling tropical Pacific sea surface temperature (SST) and the increasing Pacific trade winds^12^,^13^,^14^,^15^,^16^,^17^,^18^,^19^,^20^,^21^,^22^,^23^,^24^,^25^,^26^. This theory is supported by the successful simulation of the warming hiatus by nudging the tropical pacific SST or trade winds relative to observations^14^,^17^,^19^. The other suggests that the warming hiatus is accompanied by increasing heat uptake in global deep oceans^27^,^28^,^29^,^30^,^31^. This extra heat, which originates from a positive radiative imbalance at the top of the atmosphere (TOA), is reserved in the deep oceans instead of warming the Earth’s skin^32^,^33^,^34^,^35^,^36^. Note that both aforementioned hypotheses indeed include an enhancing greenhouse effect in which more heat is captured by the Earth–atmosphere system. The main difference between them is how this additional energy is prevented from warming the Earth’s surface.

The variation of *T*_*s*_ is commonly influenced by changes in the greenhouse effect. In theory, an enhanced (reduced) greenhouse effect will accelerate (decelerate) the upward tendency of *T*_*s*_. However, less discussion has addressed whether the Earth’s greenhouse effect is intensified as GHGs increase from the observational perspective. A few studies have used satellite-based TOA radiation observations to detect changes related to the greenhouse effect^37^,^38^,^39^,^40^,^41^. Harries *et al.*^38^ found that more terrestrial heat is captured by several main GHGs (e.g., CO_2_, CH_4_, and O_3_) in clear skies because the spectral brightness temperatures in their absorption bands used to measure the upwelling thermal energy were significantly reduced. However, their experimental evidence of an enhancing greenhouse effect was largely biased because the influences of water vapor and clouds, which contribute approximately 75% of the total effect, were not included^42^. In contrast, Raval and Ramanathan^37^ employed a parameter (*G*_*a*_) to quantify the magnitude of the atmospheric greenhouse effect including all potential contributors. *G*_*a*_ is the residual obtained by subtracting the TOA outgoing longwave radiation (OLR) from the surface upwelling longwave radiation (SULR). This parameter measures the vertically integrated greenhouse effect in the entire atmosphere and enters directly into the basic equations describing the climate. Furthermore, Cess and Udelhofen^43^ reported a significant decreasing tendency of normalized *G*_*a*_ (Δ*G*_*a*_ = *G*_*a*_/SULR) for the 40°S to 40°N domain between 1985 and 1999 based on measurements of the TOA energy budget and Earth’s surface temperature. They attributed this downward trend of the greenhouse effect to a notable reduction in cloud cover^43^.

Whether the observational greenhouse effect is intensified during the warming hiatus period remains unclear. With the steady rise of anthropogenic GHG concentrations, does the heat trapped and then re-emitted to the surface by the atmosphere also increase? In addition, the change of the Earth’s surface temperature has been shown down to be non-uniform in different regions and different sub-periods during recent decades^44^. Does the greenhouse effect have some spatial or temporal characteristics similar to those in *T*_*s*_? Thus, the primary goal of this study is to investigate the spatiotemporal evolution of the greenhouse effect to better evaluate its potential impact. In this work, the monthly gridded *G*_*a*_ between 1979 and 2014 is estimated from the High-resolution Infrared Radiation Sounder (HIRS) OLR climate dataset^45^ provided by the National Center for Environmental Information (NCEI) and the HadCRUT4 surface air temperature (*T*_s_) dataset^46^ provided by the Climatic Research Unit (CRU). The SULR is calculated using the blackbody radiation law (SULR = *σT*_s_^4^, where *σ* is the Stefan–Boltzmann constant) of Raval and Ramanathan^37^. The monthly gridded surface greenhouse effect parameter (*G*_*s*_), which is defined as the downwelling longwave radiation (*F*↓) at the Earth’s surface by Boer^47^, is also obtained using a radiative transfer model from the National Aeronautics and Space Administration (NASA) Clouds and the Earth’s Radiant Energy System (CERES) Energy Balanced And Filled (EBAF) product^48^.

## Results

**Figure 1:**
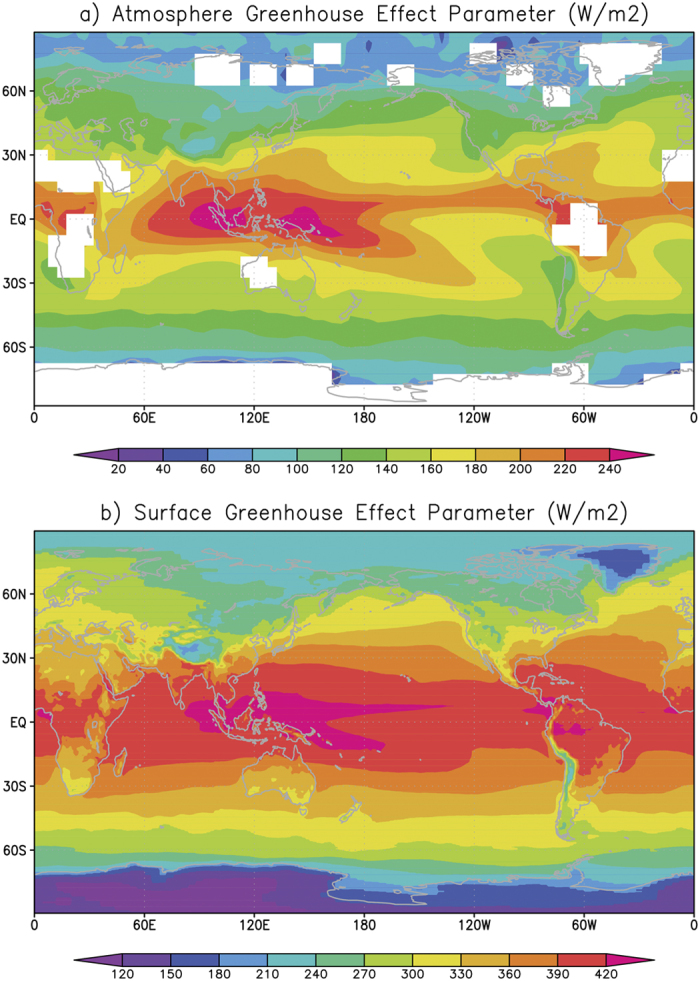
Spatial distributions of climatological averaged greenhouse effect parameter (*G*; unit: W m^−2^) on a 5° by 5° box between 2003 and 2014. (**a**,**b**) refer to the atmospheric and surface greenhouse effect parameters (*G*_*a*_ and *G*_*s*_), respectively. The maps were generated by the Grid Analysis and Display System (GrADS; http://www.opengrads.org/doc/wind32-v1/) version 1.90-rc1.

The radiative warming effects of the atmosphere and the surface in the infrared range can be described by *G*_*a*_ and *G*_*s*_^47^, whose climatological means are 158 W m^−2^ and 345 W m^−2^, respectively, from 2003 to 2014. *G*_a_ represents the ability of the atmosphere to trap approximately 40% of the longwave radiation emitted by the Earth’s surface (399 W m^−2^). *G*_*s*_ indicates the energy sent by the atmosphere to the surface to heat the Earth. Nearly half of *G*_*s*_ comes from *G*_*a*_, and the rest comprises the solar incidence, sensible and latent heat absorbed by the atmosphere^49^. Figure 1 represents the spatial patterns of the estimated mean *G*_*a*_ and *G*_*s*_ between 2003 and 2014.Although *G*_*a*_ and *G*_*s*_ are both spatially inhomogeneous, they share similar spatial distributions. First, on average, both *G*_*a*_ and *G*_*s*_ decrease with increasing latitude. The zonal means of *G*_*a*_ (*G*_*s*_) are 189 W m^−2^ (394 W m^−2^) and 90 W m^−2^ (231 W m^−2^) in the tropics (30°S–30°N) and polar zones (90°S–60°S and 60°N–90°N), respectively. The latitudinal patterns of *G*_*a*_ and *G*_*s*_ are mostly caused by the zonal distribution of the atmospheric water vapor content, which is the most important contributor to the greenhouse effect^42^. The wetter atmosphere at low latitudes thus absorbs more terrestrial radiation than the drier atmosphere at high latitudes. The surface condition is another important contributor to the *G*_*s*_ distribution. The wetter and warmer surface in the tropics provides greater latent and sensible heat to the atmosphere, which is included in *G*_*s*_^47^. Second, because more atmospheric and surface moisture is found at sea than on land, on average, the oceanic *G*_*a*_ and *G*_*s*_ (162 W m^−2^ and 358 W m^−2^) are slightly larger than the terrestrial values (148 W m^−2^ and 312 W m^−2^). When the large proportion of oceans covering the Earth’s surface is considered, the oceanic *G*_*a*_ (*G*_*s*_) contributes more than three-quarters of the global total *G*_*a*_ (*G*_*s*_). Moreover, nearly half of the global greenhouse effect is attributed to *G*_*a*_ (*G*_*s*_) over the tropical oceans. Third, either *G*_*a*_ or *G*_*s*_ displays a meridional heterogeneity at the tropics. On the one hand, larger *G*_*a*_ and *G*_*s*_ (above 220 W m^−2^ and 420 W m^−2^, respectively) are found in the Indo-West Pacific, Amazon and East Africa. This pattern coincides with the location of the tropical monsoons^50^ that often deliver persistent convection. In the monsoon-prone areas, a stronger greenhouse effect is induced by the wetter and cloudier atmosphere and by the moist surface. On the other hand, the *G*_*a*_ over the East Pacific and East Atlantic is relatively low (below 180 W m^−2^) because these areas are generally controlled by persistent subsidence and have dry and cloudless atmospheres.

**Figure 2:**
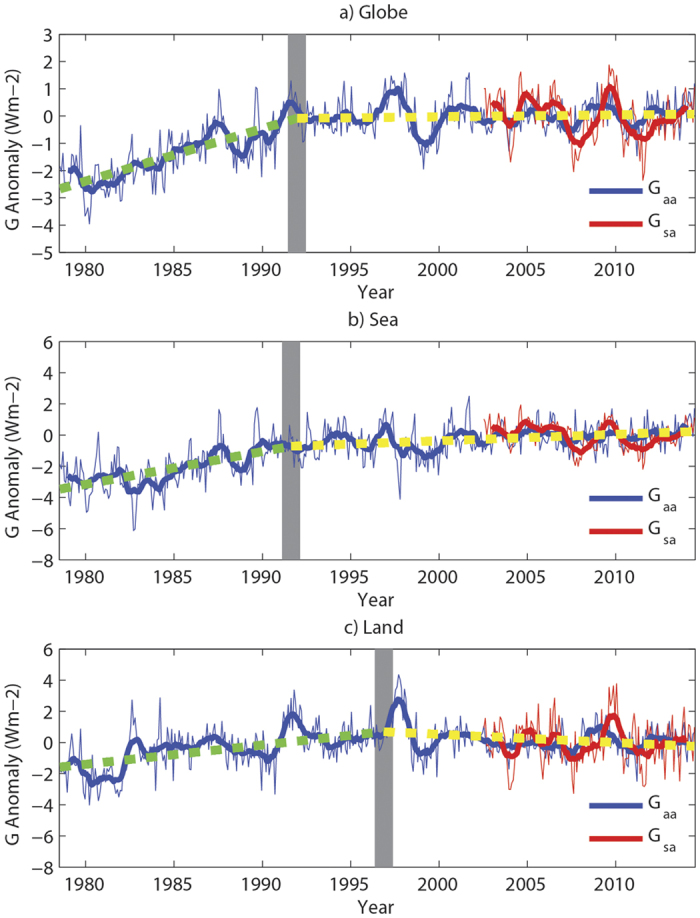
Monthly variations of the areal averaged atmospheric and surface greenhouse effect parameter anomalies (*G*_*aa*_ and *G*_*sa*_) from 1979 to 2014 for the (**a**) globe, (**b**) sea and (**c**) land. *G*_*aa*_ and *G*_*sa*_ are represented by blue and red lines, respectively. Thin and thick solid lines indicate the monthly and 12-month moving averaged series, respectively. Vertical, thick, gray lines represent the break points of trends using the break function regression (see Methods). Green and yellow dashed lines refer to linear trend lines before and after the break points, respectively. The figure was plotted using MATLAB software.

Based on the climatological (2003–2014) means of *G*_*a*_ and *G*_*s*_, the long-term variations of their anomalies (*G*_*aa*_ and *G*_*sa*_) can be obtained (Fig. 2). Because of the shorter period of the CERES EBAF product, the areal averaged *G*_*sa*_ is represented only between 2003 and 2014 in Fig. 2 but shows no notable trend over the globe, sea or land. Thus, the surface greenhouse effect has not been strengthened in the last decade. The temporal variations of *G*_*sa*_ and *G*_*aa*_ are highly correlated over the globe, sea and land in 2003–2014, with all correlation coefficients above 0.40 and significant at the 0.01 level based on Student’s *t*-test. By contrast, *G*_*aa*_ can be obtained from 1979 to 2014 because of the longer instrumental observations of *T*_*s*_ and OLR. The most obvious feature is that the decadal trends of the global averaged *G*_*aa*_ are not uniform throughout the period (Fig. 2a). In the 1980 s, a significant increasing *G*_*aa*_ tendency exists with a linear estimate of 0.19 W m^−2^ yr^−1^. However, this uprising trend pauses starting in circa 1992, when *G*_*aa*_ begins to slightly decrease at a rate of −0.01 W m^−2^ yr^−1^. This statistically non-significant trend indicates that the enhancing global atmospheric greenhouse effect is slowed down. Moreover, the atmospheric greenhouse effect hiatus can be found over both sea and land (Fig. 2b–c). Because the global total atmospheric greenhouse effect is largely controlled by the atmosphere over the oceans, the temporal variation of the averaged *G*_*aa*_ at sea is similar to the global value (Fig. 2b). The tendency of the averaged *G*_*aa*_ over the oceans also abruptly changes circa 1992. The oceanic *G*_*aa*_ exhibits a notable increasing trend with a rate of 0.21 W m^−2^ yr^−1^ in 1979–1991, whereas its rate of change (−0.04 W m^−2^ yr^−1^) during 1992–2014 is not statistically significant. By contrast, although a sudden change in the *G*_*aa*_ tendency is observed overland, the breakpoint is approximately 5 years later than that of the oceanic *G*_*aa*_ (Fig. 2c). The terrestrial *G*_*aa*_ trends are 0.12 W m^−2^ yr^−1^ and 0.05 W m^−2^ yr^−1^ before and after 1997, respectively.

Because *G*_*a*_ is jointly determined by the longwave radiation at the surface and the TOA, the *T*_*s*_ and OLR evolutions are employed to discuss the formation of the global atmospheric greenhouse effect hiatus (Fig. S1). Here, the time period is divided into three 12-year subperiods (1979–1990, 1991–2002 and 2003–2014). The first break point is used to separate the varying long-term global *G*_*aa*_ behavior in Fig. 2a. The second break point represents the beginning of the global warming pause because the increasing global averaged *T*_*s*_ tendency slowed down in the early 21^st^ century^15^. In the first subperiod (1979–1990), the increasing *T*_*s*_ leads to a remarkable uprising trend in the global averaged SULR anomaly of 0.07 W m^−2^ yr^−1^, whereas the global averaged OLR anomaly exhibits a significant decreasing trend of −0.10 W m^−2^ yr^−1^. Both of these behaviors enhance the atmospheric greenhouse effect, as indicated by an increase in *G*_*aa*_. However, in the following subperiod, the rates of change of the SULR and OLR anomalies are both significantly positive. The former (0.15 W m^−2^ yr^−1^) is comparable to the latter (0.14 W m^−2^ yr^−1^). Therefore, their contributions to the atmospheric greenhouse effect nearly cancel each other out. As a result, an unchanged global averaged *G*_*aa*_ is shown during 1991–2002. In the last subperiod, the global averaged SULR anomaly remains trendless (0.02 W m^−2^ yr^−1^) because *T*_*s*_ stops rising. Meanwhile, the long-term change of the global averaged OLR anomaly (−0.01 W m^−2^ yr^−1^) is also not statistically significant. Thus, these two phenomena result in a trendless *G*_*aa*_.

**Figure 3:**
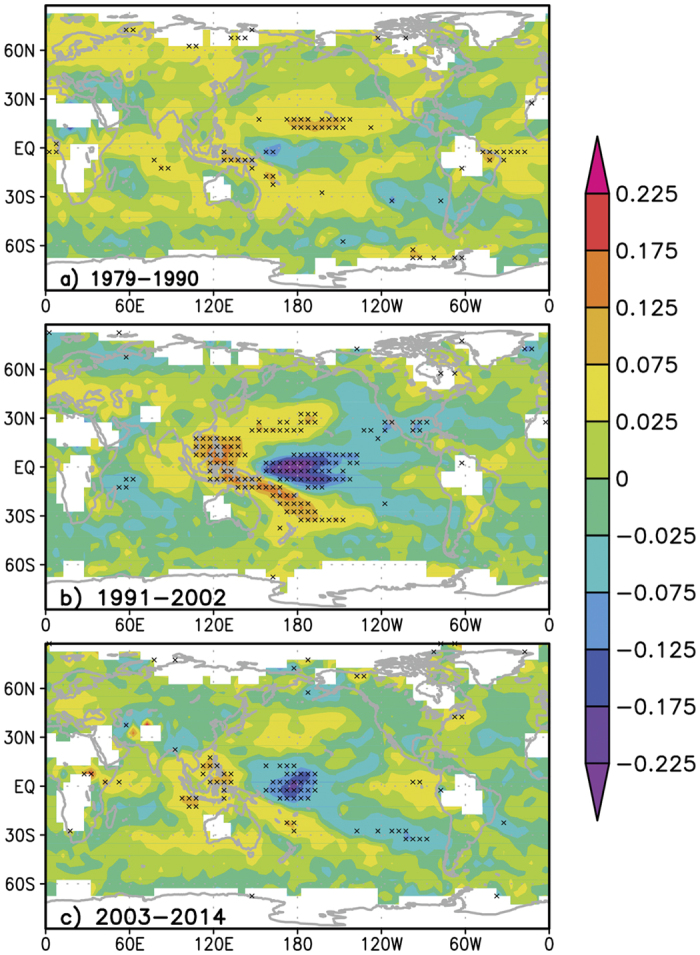
Spatial structures of the atmospheric greenhouse effect parameter anomaly (*G*_*aa*_) trend on a 5° by 5° box using the least-squares approach during three subperiods: (**a**) 1979–1990, (**b**) 1991–2002, and (**c**) 2003–2004. Regions with a significant tendency (at the 0.05 confidence level based on the *F*-test) are crossed. Maps were generated by GrADS (http://www.opengrads.org/doc/wind32-v1/) version 1.90-rc1.

Furthermore, the trends of *G*_*aa*_ are spatially inhomogeneous during individual subperiods (Fig. 3). *G*_*aa*_ increases the most over the central North Pacific with a tendency of approximately 0.12 W m^−2^ yr^−1^ in 1979–1990 (Fig. 3a). Significant upward *G*_*aa*_ trends are also found at the tropical Atlantic and the high latitudes of Eurasia. By contrast, almost no regions exhibit a significant downward *G*_*aa*_ trend. This finding explains why the global averaged *G*_*aa*_ increases during this period. Similar to the previous period, an uprising *G*_*aa*_ trend is found over the central North Pacific from 1991 to 2002 with a reduced rate (Fig. 3b). Meanwhile, *G*_*aa*_ increases by substantially more in the western tropical Pacific, where the largest tendency (0.18 W m^−2^ yr^−1^) is found, and in the central South Pacific. However, a remarkably decreasing *G*_*aa*_ trend (−0.27 W m^−2^ yr^−1^) exists over the central tropical Pacific, indicating a weakened atmospheric greenhouse effect in this area, which largely offsets the warming effect in the aforementioned surrounding regions. As a result, a trendless global averaged *G*_*aa*_ is displayed between 1991 and 2002 (Fig. 2). During the latest subperiod (2003–2014), the spatial pattern of the change in *G*_*aa*_ is quite similar to that in 1991–2002, but the proportion of regions with significant *G*_*aa*_ tendencies is significantly reduced (Fig. 3c). Although the maximum upward and downward *G*_*aa*_ tendencies also appear over the western tropical Pacific and the central tropical Pacific, respectively, the increasing trend is nearly absent in the extratropics. Again, no significant trend of the global averaged *G*_*aa*_ is found from 2003 to 2014 (Fig. 2) because the enhanced warming effect over the western tropical Pacific is largely counteracted by the weakened warming influence on the central tropical Pacific.

**Figure 4:**
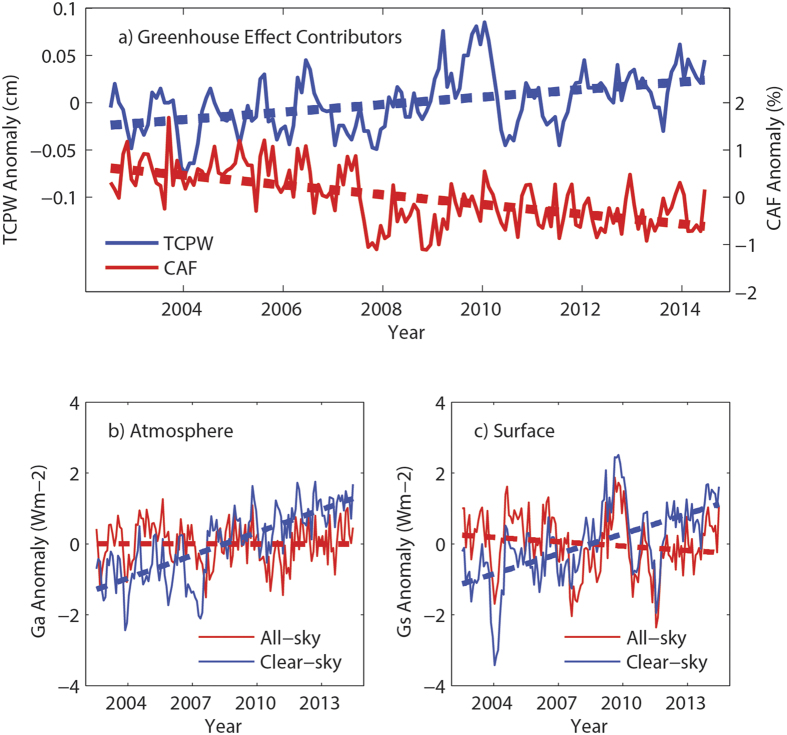
(**a**) Monthly variations of the global averaged TCPW (unit: cm) and CAF (unit: %) anomalies between 2003 and 2014. Dashed lines are the linear trend lines obtained by the least squares method. (**b**) Monthly variations of the atmospheric greenhouse effect parameter anomaly (*G*_*aa*_; unit: W m^−2^) from 2003 to 2014 for all-sky (red lines) and clear-sky (blue lines) conditions. Dashed lines are the linear trend lines obtained by the least squares method. (**c**) Same as (**b**) but for the surface greenhouse effect parameter anomaly (*G*_*sa*_; unit: W m^−2^). The figure was plotted using MATLAB software.

The results above indicate that the notably downward *G*_*aa*_ tendency over the central tropical Pacific indeed plays an important role in inducing the greenhouse effect hiatus since the 1990 s. What causes this decreasing *G*_*aa*_? The variation of the greenhouse effect is substantially influenced by its contributors, including water vapor, clouds, and GHGs^42^. GHG concentrations have risen steadily during recent decades^1^. The variations of metrics related to the other two contributors are given in Fig. 4a and are based on the CERES-EBAF products between 2003 and 2014. The total column precipitable water (TCPW) anomaly significantly increases at a rate of 0.44 cm yr^−1^. However, the cloud area fraction (CAF) anomaly is reduced by −0.60% yr^−1^, which is consistent with the decreasing cloud activity described in previous publications^51^. Therefore, although the greenhouse effect can be enhanced by increasing GHGs and water vapor in the atmosphere, it can be weakened by decreasing clouds. If these two actions offset each other, a hiatus of the global greenhouse effect will result. To confirm this, the variations of *G*_*aa*_ and *G*_*sa*_ in all-sky conditions are compared with those in clear-sky conditions in Fig. 4b,c. The clear-sky atmospheric and surface greenhouse effect parameters increase significantly at rates of 0.22 W m^−2^ yr^−1^ and 0.19 W m^−2^ yr^−1^, respectively. However, the atmospheric and surface greenhouse effect parameters both become trendless when clouds are considered. Moreover, the spatial pattern of the CAF anomaly trend (Fig. S2) is very similar to that of the *G*_*aa*_ trend (Fig. 3c) during 2003–2014. Cloud activity becomes less active over the central tropical Pacific, whereas it is enhanced over the western and eastern tropical Pacific. Overall, the downward tendency of clouds is the dominant contributor to the greenhouse effect hiatus.

Interestingly, the spatial structure exhibits a seesaw pattern between the central tropical Pacific and the western/eastern tropical Pacific in both the *G*_*aa*_ tendency and CAF anomaly trend from 2003 to 2014 (Fig. 3c, Fig. S2). This pattern is similar to that of the composited SST anomaly in strong La Niña events^51^,^52^. Further, the decreasing *G*_*aa*_ trend can result from La Niña events occurring more frequently in the last two decades. The Niño 3.4 SST anomaly shows no significant tendency between 1979 and 1990, whereas it decreases remarkably after 1990 at a rate of −0.028 °C yr^−1^ (Fig. S3). Notably, the first half of the downward Niño 3.4 SST anomaly trend is much larger than the second half. This finding is consistent with a stronger decreasing *G*_*aa*_ trend over the central tropical Pacific during 1991–2002 (Fig. 3). Strong La Niña events are associated with strong anomalous cooling and suppressed convection in the central tropical Pacific^52^,^53^. Therefore, this La Niña-related phenomenon can reduce atmospheric water vapor content and cloud volume and further weaken the greenhouse effect over the central tropical Pacific.

## Discussion

The Earth’s environment is suitable for life because of the greenhouse effect. Our planet has become increasingly warm since the Industrial Revolution because of the increased GHG emissions, which greatly enhance the greenhouse effect. However, the uprising rate of the Earth’s *T*_*s*_ has slowed down in recent years. Whether this global warming pause is accompanied by a hiatus of the greenhouse effect is investigated in this study. The regional and global greenhouse effects are quantitatively estimated from reliable *T*_*s*_ observations and consistent OLR satellite products in 1979–2014. Although the change of OLR is theoretically in accordance with that of the tropospheric temperature according to the Stefan–Boltzmann Law, in reality, their relationship is more complex^54^. Different tendencies of OLR and *T*_*s*_ can be seen in different periods, leading to an atmospheric and surface greenhouse effect hiatus since the early 1990 s. This pause exists not only over the oceans but also over the continents. Further analysis indicates that this hiatus is very likely a result of the occurrence of more La Niña events after 1992. In the strong La Niña phase, both the atmospheric water vapor and the cloud volume are greatly reduced over the central tropical Pacific, amplifying the regional weakened greenhouse effect. Therefore, *G*_*aa*_ decreases significantly during 1992–2014 over the central tropical Pacific, which offsets the upward *G*_*aa*_ tendencies occurring elsewhere.

**Figure 5:**
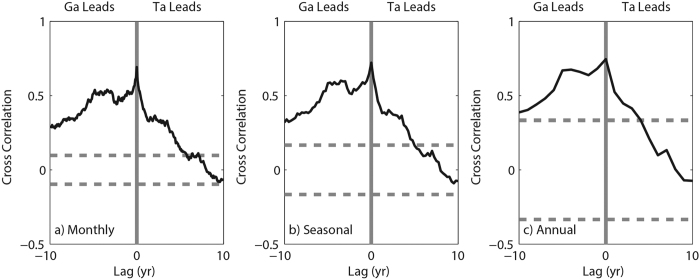
Cross-correlation coefficients between the global averaged atmospheric greenhouse effect parameter anomaly (*G*_*aa*_) and the global mean surface temperature anomaly (*T*_*a*_) on three time scales: (**a**) monthly, (**b**) seasonal and (**c**) annual. The horizontal dashed line refers to the significance at the 0.05 level. Positive (negative) lags indicate that *T*_*a*_ leads (trails) *G*_*aa*_. The figure was plotted using MATLAB software.

Interestingly, the atmospheric greenhouse effect hiatus occurs ahead of the global warming slowdown. Does the former lead to the latter? To answer this question, the cross-correlation coefficients between the *G*_*aa*_ and the *T*_*s*_ anomaly (*T*_*a*_) on different timescales are given in Fig. 5. The simultaneous correlations are largest on the whole timescales, which likely indicates a positive feedback between *T*_*s*_ and the greenhouse effect. By contrast, the secondary maximum consistently appears with a lag of approximately 5 years. Moreover, larger and more significant correlations are found when *G*_*aa*_ leads *T*_*a*_ than when *G*_*aa*_ trails *T*_*a*_. Thus, the variability of *T*_*a*_ may depend on the foregoing change of *G*_*aa*_. In conclusion, the pause of the greenhouse effect since the 1990 s may be one of the reasons for the global warming hiatus starting in the early 2000 s.

It is well accepted that the recent global warming slowdown is attributable to the joint effect of internal natural variability and external forcing^12^. In general, the warming hiatus is mainly driven by internal variability such as a negative phase of the IPO as well as a more La Niña-dominated state, with a minor external contribution^8^. However, a recent study found that the phase of the IPO could be modulated by anthropogenic aerosols, in which case external forcing was attributed to be the primary factor decelerating global warming^55^. By contrast, this study is not focused on the potential causes of different IPO or ENSO phases. Instead, we represent an alternative pathway of internal variability driving the warming slowdown. A La Niña-like state suppresses convection in the tropical central Pacific and concomitantly reduces cloud coverage. Consequently, a zero-trend greenhouse effect is achieved under the balance of its primary contributors (e.g. water vapor, clouds, and GHGs). Finally, the hiatus of the greenhouse effect-driven warming leads to the recent global warming slowdown, in which the atmosphere traps (emits) near constant heat from (to) the surface.

## Methods

### Surface temperature (*T*_*s*_)

The global monthly absolute *T*_*s*_ records are derived by combining the temperature anomaly and the base temperature with a spatial resolution of 5°long × 5°lat from 1979 to 2014. The combined land and marine *T*_*s*_ anomalies relative to the base period 1961–1990 are provided by the joint work of the CRU of the University of East Anglia and the Hadley Centre of the UK Met Office^56^. The corresponding absolute *T*_*s*_ for the aforementioned base period is given by Jones *et al.*^57^.

### HIRS OLR

The global monthly product of OLR at the TOA is obtained from the Climate Data Record (CDR) program organized by the National Oceanic and Atmospheric Administration (NOAA) in 1979–2014^45^. These consistent, long-term OLR records are derived using the radiance observations from the HIRS onboard the NOAA Television Infrared Observation Satellite (TIROS)-N series and the Eumetsat MetOp-A satellites. The original gridded 2.5° × 2.5° OLR data are interpolated on a 5° by 5° box.

### NASA CERES EBAF satellite products

The monthly OLR and surface downwelling longwave radiation in all-sky and clear-sky conditions between 2003 and 2014 are provided by the NASA CERES EBAF product^48^. The monthly CAF and TCPW are from the same product. The original gridded 1° × 1° data are all interpolated on a 5° by 5° box.

### Atmospheric greenhouse effect parameter (*G*_*a*_)

The monthly *G*_*a*_ values are calculated by 
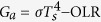
on each 5° by 5° grid^42^, where *σ* is the Stenfan–Boltzmann constant (5.67 × 10^−8^ W m^−2^ K^−4^). *G*_*a*_ represents the difference between the heat emitted by the Earth’s surface and the longwave radiation escaping from the TOA. The former energy is estimated based on the assumption of blackbody radiation; thus, the emissivity (*ε*) is close to one for every underlying surface. This assumption is confirmed by the global surface emissivity map (Fig. S4) derived using the NASA CERES EBAF products^48^.

### Surface greenhouse effect parameter (*G*_*s*_)

The monthly *G*_*s*_ values are defined by 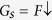 on individual 5° by 5° grids, where *F*↓ refers to the downwelling longwave radiation at the Earth’s surface.

### Break function regression

This procedure objectively estimates the change point of different tendencies in time series by combining a weighted least-squares criterion with a brute-force search and the linear trends before and after the discontinuity^58^. It is similar to the piecewise linear regression method with one change point reported in other publications^6^,^59^,^60^. The break function model for time series *X(T*), written as


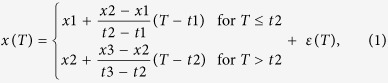


has four parameters: *x*1, *x*2, *x*3 and *t*2. The former three can be estimated using the least-squares approach when *t*2 is fixed. The break point *t*2 is determined by maximizing the explained variance. The trends before and after the break point are determined as (*x*2-*x*1)/(*t*2-*t*1) and (*x*3-*x*2)/(*t*3-*t*2), respectively.

### Trend analysis

The time series trend is estimated by the least squares method. In this study, the tendency is considered significant when it passes the *F*-test at the 0.05 level.

## Additional Information

**How to cite this article**: Song, J. *et al.* A Hiatus of the Greenhouse Effect. *Sci. Rep.* **6**, 33315; doi: 10.1038/srep33315 (2016).

## Supplementary Material

Supplementary Information
