# Supplementary material for: A Hiatus of the Greenhouse Effect
Source: Sci Rep. 2016 Sep 12;6:33315. doi: 10.1038/srep33315 (PMC5018860; doi:10.1038/srep33315)
Supplement: Supplementary Information [file srep33315-s1.pdf]

Supplementary Information of

A Hiatus of the Greenhouse Effect

By

Jinjie Song, Yuan Wang, and Jianping Tang

Second Revision

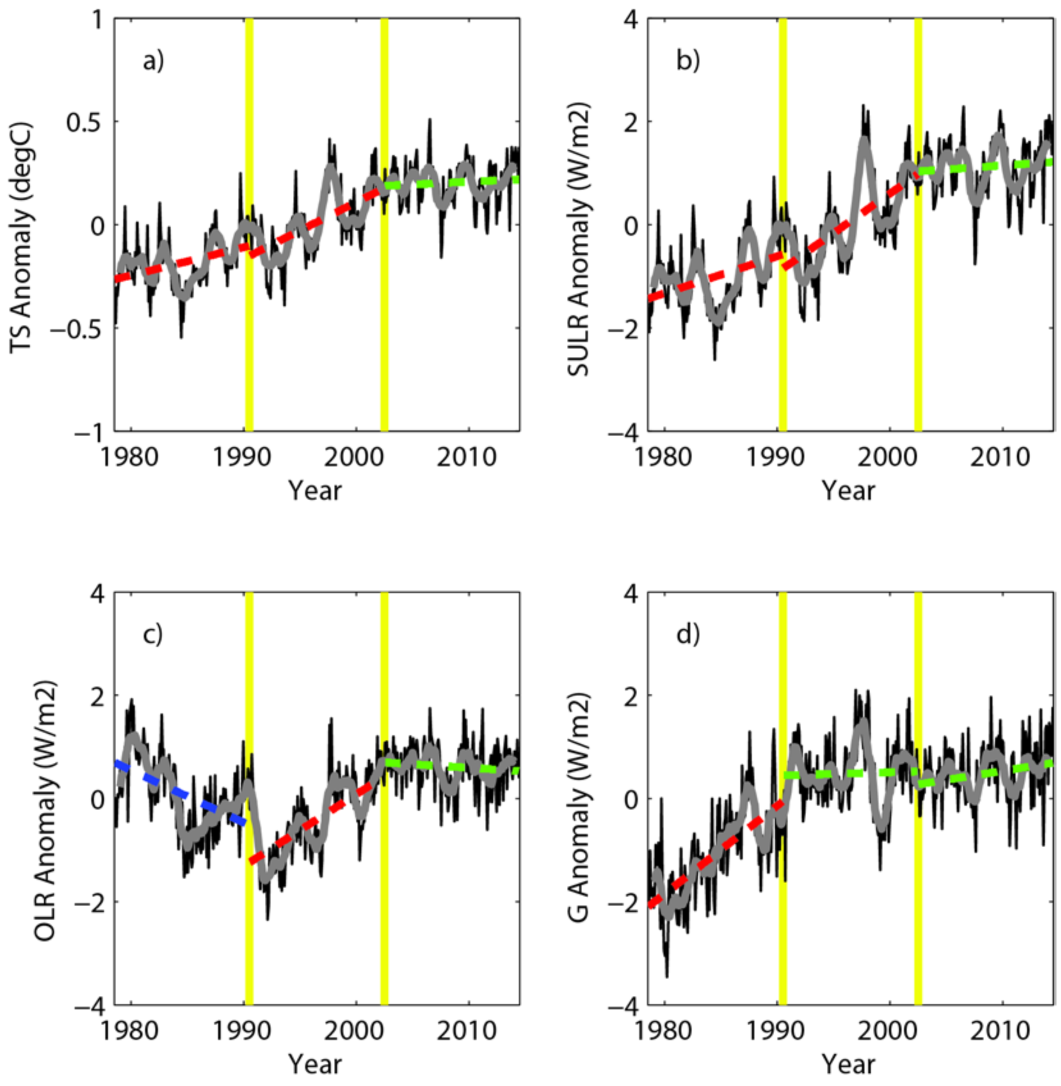

Figure S1. Monthly variations of global averaged values during 1979-2014 for (a) surface temperature anomaly, (b) surface upwelling longwave radiation anomaly, (c) outgoing longwave radiation anomaly at top of the atmosphere and (d) atmospheric greenhouse effect parameter anomaly. Red and blue dashed lines indicate significant upward and downward trends, respectively, whereas trendless variations are referred to green dashed lines. The significance is determined by the *F*-test on the 0.05 level. The figure was plotted using Matlab software.

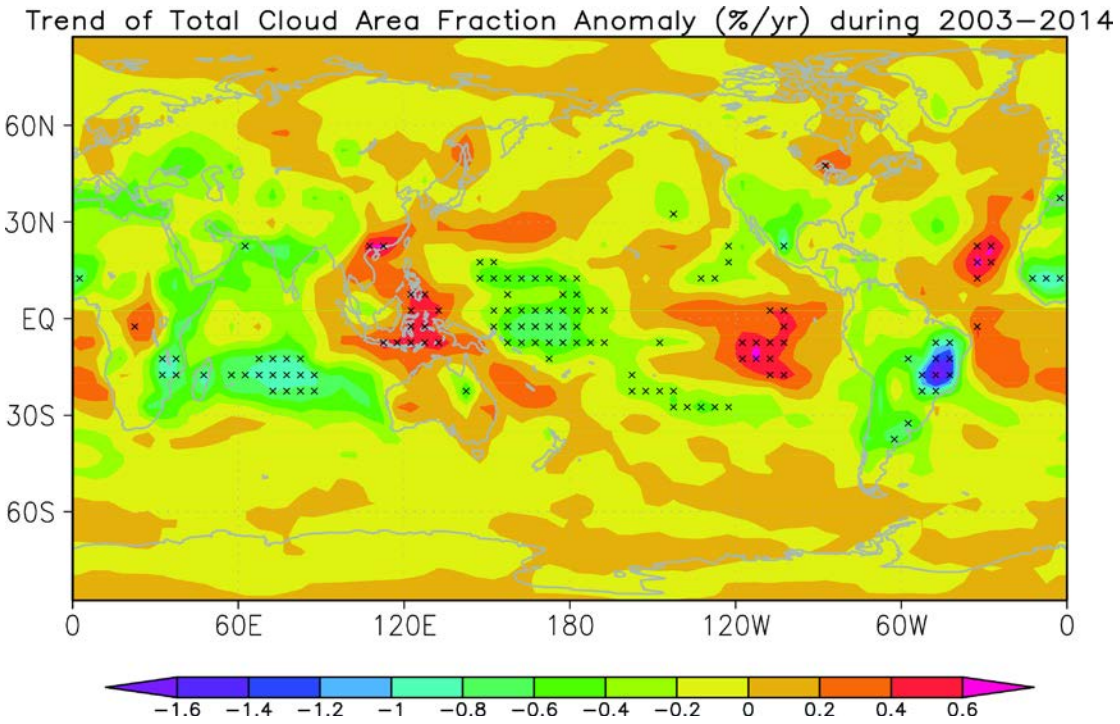

Figure S2. Spatial structure of the total cloud area fraction (CAF; unit: %) anomaly trend on a 5° by 5° box using the least-squares during 2003-2004. Regions with a significant tendency(at the 0.05 confidence level based on the *F*-test) are crossed. Maps were generated by GrADS.

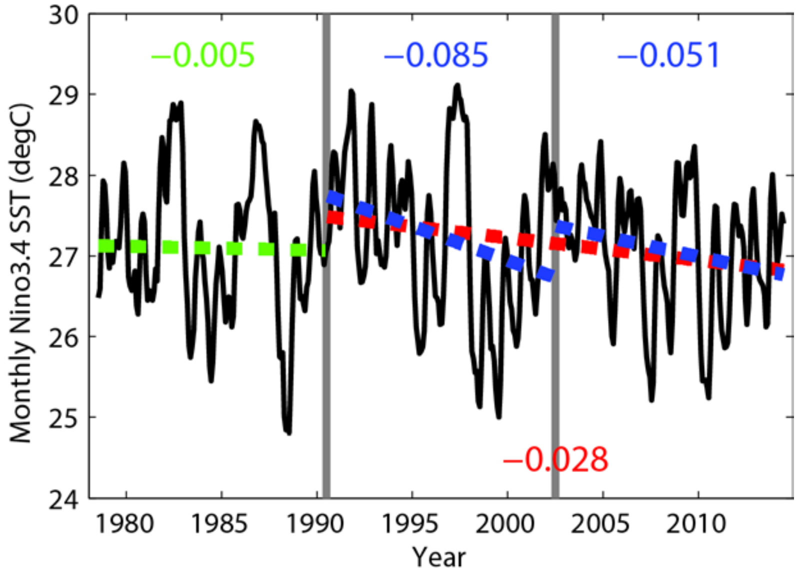

Figure S3. Monthly variation of the sea surface temperature (SST; unit: ^{0}C) anomaly over the Nino 3.4 region (5^0N~5^0S, 170^0W~120^0W) obtained from the Earth System Research Laboratory (ESRL) of the U.S. National Oceanic and Atmospheric Administration (NOAA) (url: http://www.esrl.noaa.gov/psd/data/climateindices/). Dashed lines represent linear trend lines during different periods, and colored numbers indicates the corresponding linear trend estimated by the least-squares. The figure was plotted using Matlab software.

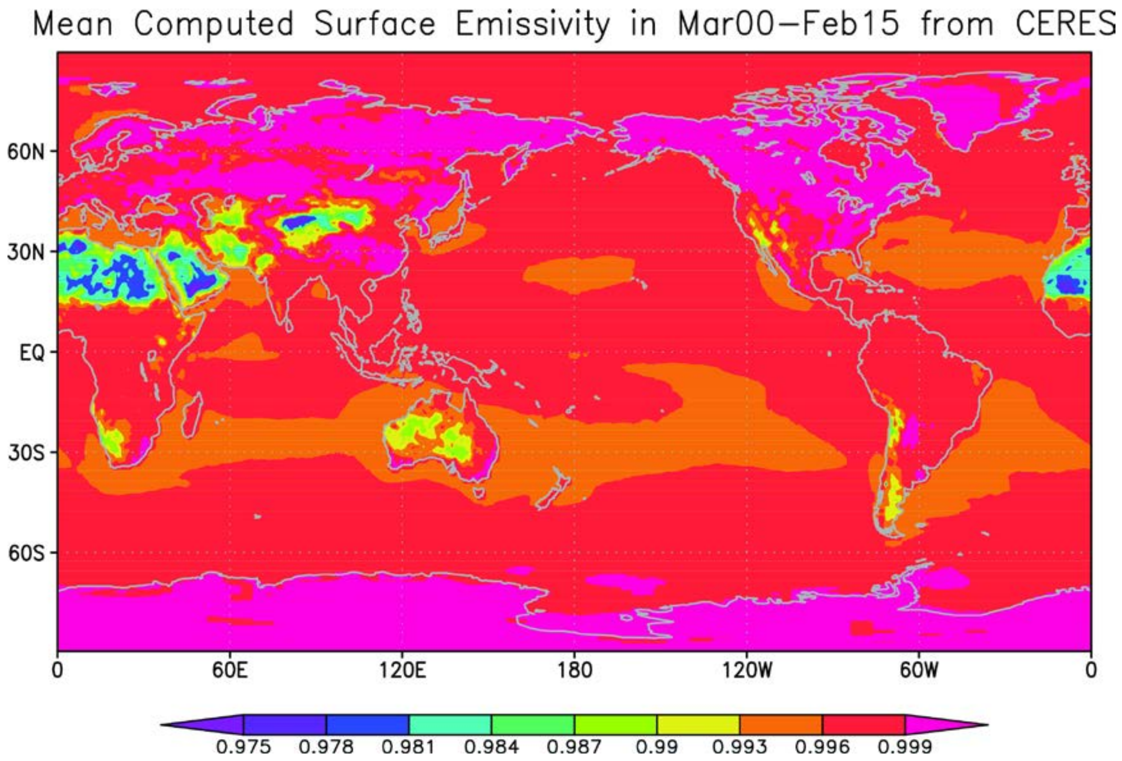

Figure S4. Spatial distribution of the averaged surface emissivity (ε) usingε=$LW_{surf}/\sigma T_{skin}^{4}$ from MAR 2000 to FEB 2015. LW_{surf} is the estimated surface upwelling flux and T_{skin} refers to the skin temperature. Both variables are provided by the CERES program (Wielicki et al., 1996). The map was generated by the Grid Analysis and Display System (GrADS; http://www.opengrads.org/doc/wind32-v1/) version 1.90-rc1.
